# F74. CLINICAL SYMPTOMS AND NOT OBJECTIVE COGNITIVE PERFORMANCE DRIVE SUBJECTIVE COGNITIVE COMPLAINTS IN PATIENTS WITH SCHIZOPHRENIA

**DOI:** 10.1093/schbul/sby017.605

**Published:** 2018-04-01

**Authors:** Jack Cotter, Kiri Granger, John Evenden, Jennifer Barnett, Michael Sand

**Affiliations:** 1 Cambridge Cognition; 2 Boehringer Ingelheim

## Abstract

**Background:**

The United States Food and Drug Administration (FDA) recommends that drugs that exert a pro-cognitive effect should be accompanied by measurable improvements in ‘real-world’ functioning. Patients with schizophrenia typically exhibit substantial impairments across a wide range of cognitive domains, representing an important target for therapeutic intervention. This has led to the development of several instruments specifically for use in this population that seek to capture the subjective experience and impact of cognitive dysfunction on daily living for use as co-primary endpoints in clinical trials. However, it remains unclear to what extent these accurately reflect objective cognitive performance.

**Methods:**

We conducted a secondary analysis of data from 413 patients with schizophrenia who participated in a multi-national randomized, double-blind, placebo-controlled trial. During the trial, participants completed two different neurocognitive test batteries, the Cambridge Neuropsychological Test Automated Battery (CANTAB) and the MATRICS Consensus Cognitive Battery (MCCB). They also completed two measures of subjective cognitive performance, the Schizophrenia Cognition Rating Scale (SCoRS) and the Patient Reported Experience of Cognitive Impairment in Schizophrenia (PRECIS). The severity of patient’s symptoms was also assessed at each time point using the Positive and Negative Syndrome Scale (PANSS). All assessments were conducted at baseline and at a 12-week follow-up assessment (end of treatment). We examined the associations between these variables using correlational and multivariable linear regression analyses.

**Results:**

Scores on each of the subjective measures of cognition were weakly correlated in the expected direction with objective measures of cognitive performance across both the CANTAB and MCCB tasks. Scores on each of the subjective cognition measures were more strongly associated with severity of symptoms as assessed using the PANSS. Multivariable regression analyses suggested that clinical symptoms accounted for significantly greater variance in subjective cognition scores than either objective cognitive performance or demographic factors.

**Discussion:**

Subjective appraisals of cognition are poor predictors of objective cognitive performance in patients with schizophrenia. The burden reported by patients on these instruments appears to be more closely associated with the severity of their clinical symptoms. This has important implications for the use of these measures as co-primary endpoints in clinical trials assessing pro-cognitive drug effects.

